# Nitride-Based Materials for Flexible MEMS Tactile and Flow Sensors in Robotics

**DOI:** 10.3390/s17051080

**Published:** 2017-05-10

**Authors:** Claudio Abels, Vincenzo Mariano Mastronardi, Francesco Guido, Tommaso Dattoma, Antonio Qualtieri, William M. Megill, Massimo De Vittorio, Francesco Rizzi

**Affiliations:** 1 Center for Biomolecular Nanotechnologies @UNILE, Istituto Italiano di Tecnologia (IIT), Arnesano (LE) 73010, Italy; claudio.abels@hsrw.eu (C.A.); vincenzo.mastronardi@iit.it (V.M.M.); francesco.guido@iit.it (F.G.); tommaso.dattoma@iit.it (T.D.); antonio.qualtieri@iit.it (A.Q.); 2 Dipartimento di Ingegneria dell’Innovazione, Università del Salento, Lecce 73100, Italy; 3 Faculty of Technology and Bionics, Rhine-Waal University of Applied Sciences, Kleve 47533, Germany; william.megill@hsrw.eu

**Keywords:** MEMS, stress-driven, aluminum nitride, silicon nitride, piezoelectric, piezoresistive, tactile sensing, flow sensing

## Abstract

The response to different force load ranges and actuation at low energies is of considerable interest for applications of compliant and flexible devices undergoing large deformations. We present a review of technological platforms based on nitride materials (aluminum nitride and silicon nitride) for the microfabrication of a class of flexible micro-electro-mechanical systems. The approach exploits the material stress differences among the constituent layers of nitride-based (AlN/Mo, Si${}_{x}$N${}_{y}$/Si and AlN/polyimide) mechanical elements in order to create microstructures, such as upwardly-bent cantilever beams and bowed circular membranes. Piezoresistive properties of nichrome strain gauges and direct piezoelectric properties of aluminum nitride can be exploited for mechanical strain/stress detection. Applications in flow and tactile sensing for robotics are described.

## 1. Introduction

Inspired by nature, where biological sensors can adapt their mechanical sensitivity to cope with large changes in the environment, flexible functional materials can be used to tune and efficiently control how mechanical information can be detected and transferred in a biomimetic sensor design. Recent developments in thin-film-based MEMS have enabled the cost-effective manufacture of these flexible functional materials.

The sensing elements of the MEMS devices we review here typically consist of deformable flexural elements, such as cantilever beams, membranes and bridges, which are suspended above a base layer. These are usually designed and fabricated with silicon as the structural material [1,2]. Multiple additional layers are added to the base using appropriate deposition techniques in order to create a controllable stress gradient across the layered structure. On release (which has to be done carefully to avoid problems due to upper layers adhering to lower ones during the etching process [3]), the stress gradient creates “out-of-plane” architectures, such as upwards-bent cantilevers or dome-shaped membranes. This approach makes it possible to design mechanical devices that are sensitive to both normal and shear forces, widening the range of applications of flexible MEMS towards tactile, pressure and flow sensing in robotics.

The flexural stiffness of the deformable elements has to be carefully designed and implemented by growth techniques in order to maximize the sensitivity of the sensor while ensuring it does not fail due to mechanical overloading. The micro-scale geometrical design made possible through modern MEMS manufacturing techniques provides the tool required to solve this apparent paradox. The thickness, width, length and curvature of the deformable element can all be determined quantitatively at design time, and the cavities left behind during the release process can provide shelter for the deformable element during periods of overload by distributing the force over the larger and stronger main substrate.

This review focuses on approaches to the design and fabrication of “stress-driven” flexible MEMS for sensing applications, providing an overview of some recent technological improvement in microfabrication of nitride-based devices (either aluminum nitride and silicon nitride) as a platform for developing a class of compliant and flexible MEMS undergoing and withstanding large deformations. In Section 2, we will introduce the materials science principles for obtaining nitride-based stress-driven microsystems. Section 3 describes the microfabrication methods for two different classes of stress-driven MEMS structures, i.e., piezoresistive upwards-bent cantilever beams and piezoelectric bowed circular membranes. In Section 4, results and application examples of bio-inspired piezoelectric tactile and piezoresistive flow sensors will be presented. Finally, conclusions are drawn in Section 5.

## 2. Materials

The realization of a stress-driven design requires a careful choice of material characteristics and an appropriate technology for material deposition. The proper choice of mechanical properties (shear modulus *G*, Young’s modulus *E* and Poisson ratio ν) and suitable design parameters (thickness *t*) can be combined to design a specific flexural stiffness for flexible MEMS elements such as membranes and beams. Embedding and overlaying thin films with different thermal and elastic properties generates internal mechanical stresses. These forces are caused by multiple factors. The mismatch of atomic sizes in the different materials or the lattice-mismatch due to thermal treatments during material deposition and device microfabrication cause an intrinsic stress, which is usually considered detrimental to MEMS purposes, as it often causes failure due to surface wrinkles [4] or to buckle-delamination [5,6] in compressive loading, or to channel cracks, which often appear in tensile loading [6]. In the “stress-driven” design approach, however, this phenomenon is instead directly exploited as an intrinsic release mechanism, which projects the suspended parts (beams and membranes) from the plane into the overlaying space.

Previous approaches to this design were based on a single layer structural element, such as a single silicon nitride layer grown by chemical vapor deposition (CVD) [7], polycrystalline silicon [8] and silicon dioxide [9]. The designed structural elements were affected by a stress gradient along their thickness, induced during their growth process. These approaches were characterized by a residual stress value, strongly related to the deposition technologies exploited to grow the single layer, and were difficult to replicate. As a consequence, it was hard to control the intrinsic stress conditions of the whole structure. A step forward came when the stressed structural element was designed as a multilayer structure. The stress control in the bent structure was obtained by carefully engineering the material stress difference among each multilayer component [10]. It was the relative difference among the internal stresses of different layers grown by CVD or epitaxial growth on a sacrificial layer that generated the required gradient in the stress profile through the multilayer thickness. If a tensile stress difference were realized in the multilayer, a controllable and repeatable upwards-bent mechanical structure could be obtained by detachment from a sacrificial or flexible layer via wet etching the layer underneath the structure away (e.g., cantilever beam fabrication), or by defining the mechanical element via adhesion detachment by stress relaxation (e.g., dome fabrication) [3].

The approach described above has been realized by exploiting nitride-based materials, such as Silicon Nitride (SiN) and Aluminum Nitride (AlN), whose intrinsic stress can be controlled via physical sputtering deposition or Low Pressure Chemical Vapor Deposition (LPCVD).

### 2.1. Silicon Nitride Stressor Layer

The careful control of silicon nitride residual stress has been studied since 1985, when Claassen et al. [11] studied the influence of the temperature, gas pressure and composition of silicon nitride thin films deposited on silicon substrates. Their findings were based on the observation of a temperature-dependent residual stress in the deposited film, ranging between tensile at high temperatures and compressive at low temperatures. More recent work has since been published on stress control of silicon nitride layers [12,13]. Silicon nitride-coated Silicon-On-Insulator (SOI) substrates are now the preferential template choice for silicon-based MEMS fabrication. This template consists of a 2 μm-thick silicon (device) layer on a 2 μm-thick Silicon dioxide (SiO${}_{2}$) layer. Both layers are placed on a silicon substrate with a Miller index of (100). This template is obtained by depositing a thermal SiO${}_{2}$ layer on a silicon substrate, followed by bonding the SiO${}_{2}$ side on another silicon substrate. The SOI substrate is completed by mechanical thinning of one of the two bonded silicon substrates to 2 μm. Finally, a stoichiometric Silicon Nitride (Si${}_{3}$N${}_{4}$) layer is grown by LPCVD on both sides of the SOI substrate. This layer has an intrinsic tensile stress of 800 MPa, developed during the thermal cycling connected with the deposition, and is enough to generate a stress-difference in a Si/Si${}_{3}$N${}_{4}$ bi-layered beam. For a beam length of 1500 μm, this stress value generates an upwards bending up to 1200 μm [14]. The placement of a piezoresistive strain gauge on the most stressed part of these mechanical elements, realized by deposition of metallic resistances or ion implantation, allows these devices to measure mechanical strain caused by fluid flow and shear forces [7,10,15].

### 2.2. Aluminum Nitride Stressor Layer

Aluminum nitride thin films can be grown by sputtering with excellent polycrystalline quality on different substrates including dielectrics, semiconductors and metallic layers, as well as on flexible substrates, such as polymers and polyimides [16,17,18,19,20,21,22,23,24]. Due to its good electromechanical properties and low temperature growth technique (below 400 ${}^{\circ }C$) in the sputtering chamber, AlN is suitable for the fabrication of stress-driven MEMS devices [25]. Previous studies faced the problem to control the built-in stress and the piezoelectric properties of AlN thin films deposited by reactive magnetron sputtering [16,17,22]. It was found that stress and piezoelectricity were very much dependent on the growth conditions (e.g., pressure, gas compositions, flow rates, bias voltage and underlying layers). Dubois et al. [16] investigated aluminum nitride thin films on polycrystalline metal electrodes. The typical columnar microstructure was obtained at a substrate temperature of 400 ${}^{\circ }C$ without correlation with the crystalline mismatch between AlN and the electrode. In parallel, the internal stress was found to be insensitive to the electrode material, but strongly dependent on the sputtering parameters and in particular to ion energy bombardment, which was able to generate step transitions from a tensile state to a compressive state. In contrast, the piezoelectric properties were found dependent on both electrode material and sputtering conditions. For example, platinum electrodes yielded better piezoelectricity values than aluminum and titanium, while particles deposited on the electrode were found to bear a certain amount of energy to generate a film with a high piezoelectric constant (d${}_{33}$). Therefore, a trade-off between piezoelectricity, residual stress and crystalline quality of aluminum nitride should be found in order to increase the electromechanical coupling factor. Iborra and coworkers [17] found out that it was possible to control the negative effects of ionic bombardment (residual stress in first instance) by increasing the processing pressure in order to generate a more superficial interaction of ions with the growing surface and to obtain the worthwhile c-axis orientation and the columnar structure with a high value of the electromechanical coupling factor. Finally, a study of Jackson et al. [22] focused on the role of lattice mismatch with the substrate, validating the hypothesis that its reduction resulted in higher quality of AlN, regardless on which metal electrode aluminum nitride was deposited. In summary, a careful control of the energy supplied to ions allows for growing a pure c-axis orientation with a good piezoelectric response. Moreover, stress could range from strong compression to high tension by controlling the crystal mismatch between the underlying growth substrates.

In dependence of which stress-driven-based mechanical element will be fabricated, a proper substrate needs to be chosen. In order to obtain the control of residual stress in a cantilever structure, a silicon dioxide/silicon template can be used. Flexible beam fabrication was demonstrated on a silicon template made up of a 200 nm-thick silicon dioxide layer, which is thermally grown on a (100) silicon substrate [26]. A 600 nm-thick AlN layer upon a 100 nm-thick Molybdenum (Mo) layer is grown by DC magnetron sputtering. For a 200 μm-long AlN/Mo beam, this stress gradient generates a tip height of approximately 150 μm [26]. Control of AlN residual stress by sputtering was obtained on polyimide [19], commercial photoresist SU-8 (MicroChem Corp., Westborough, MA, USA) [24] and parylene [23] flexible and soft substrates for flexible mechanical elements. For dome fabrication, a 25 μm-thick polyimide layer is laminated on a silicon substrate, and subsequently, a sputtering growth process follows with the deposition of 120 nm-thick Mo followed by an 800–1000 nm-thick AlN layer. In an analogous manner, AlN/Mo-based multilayers grown on a flexible polyimide substrate release tensile stress by an upward deflection reaching approximately 40 μm for a diameter of 350 μm [27]. Consequently, structural elements based on AlN/Mo or Mo/AlN/Mo multilayers can be used for bent structures, such as beams and membranes [26,27,28].

### 2.3. Piezoelectric Properties of Aluminum Nitride

The piezoelectric properties of aluminum nitride transform mechanical bending (as the response of the structure to external forces) into electrical signals, turning the bent structure into an efficient transducer. AlN possesses a number of key characteristics that make it very attractive when compared to other piezoelectric materials. ZnO and lead zirconate titanate (PZT) are well-known piezoelectric materials, which are limited in their application by the complexity and compatibility of their fabrication process (contamination risks in standard CMOS lines). Organic polymers such as Polyvinylidene Fluoride (PVDF) and its copolymer Poly(Vinylidene Fluoride-co-Trifluoroethylene) (P(VDF-TrFE)) have been widely used as tactile sensor piezoelectric materials [29,30,31]. PVDF is tough, extremely flexible, has a fast dynamic response and is readily available in the form of thin films [32]. PVDF is however difficult to involve in conventional microfabrication processes as it needs mechanical stretching to achieve the piezoelectric polar β-phase and high voltage poling to force the alignment of the internal dipoles.

Poly-crystalline inorganic aluminum nitride thin films can be obtained and easily deposited by reactive sputtering at relatively low temperature (from 160 ${}^{\circ }C$ up to 250–300 ${}^{\circ }C$) [19,33] and directly grown with a very high degree of crystallinity even on plastic and flexible polymeric substrates, as polyimide. Moreover, it does not require poling or post-annealing procedures (as for PZT and PVDF) to exhibit and enhance its piezoelectric properties, since it is not a ferroelectric material. The high chemical stability of AlN allows it to be used in humid environments. Its high melting point allows AlN to withstand harsh environments, including temperatures above 500 ${}^{\circ }C$, and its mechanical properties allow it to withstand high pressures. In spite of its piezoelectric coefficients, which are lower than other piezoelectric thin films (d${}_{33}\approx$ 4.9 $pC\;N{}^{-1}$ and d${}_{31}\approx$ −1.9 $pC\;N{}^{-1}$), it has a low dielectric constant ($\epsilon_{r}\approx$ 9–11) and low dielectric losses as a consequence, which results in an improved electromechanical coupling coefficient. Furthermore, AlN does not introduce contaminants into the CMOS micro-technology process, making it completely compatible with standard microfabrication technologies. It is also a nontoxic and, hence, highly bio-compatible material, as demonstrated by Jackson et al. [34]. Finally, the possibility to deposit extremely thin and flexible films of AlN on polymeric substrate together with very good electromechanical properties makes this material an excellent choice for the development of flexible tactile sensors. The strain gradient due to the bending of stress-driven structures and the resulting flexoelectric effect (the property of some dielectric solids to exhibit a linear relationship between the spontaneous electrical polarization and the gradient of mechanical strain) in non-ferroelectric aluminum nitride are responsible for an additional polarization, leading to an enhancement of the transduction properties of the material [35,36].

The integration of flexible polyimide with AlN thin film deposited by sputtering techniques has made possible the development of a wide range of flexible and compliant devices, from piezoelectric tactile sensors to piezoelectric micromachined Ultrasonic Transducers (pMUTs) and micro power generators, all based on stress differences between the constituent layers. By exploiting the quasi-unavoidable mechanical stress in these films due to the lattice mismatch and difference in thermal expansion coefficients of film and substrate, the sensory performance of structures such as upwards-bent cantilever beams and domed circular membranes can be improved.

## 3. Methods

This section describes the microfabrication process of two different classes of stress-driven MEMS structures, namely piezoresistive upwards-bent cantilever beams and piezoelectric tactile membranes.

### 3.1. Microfabrication of Piezoresistive Upwards-Bent Cantilever Beams

In the past, different stressor layers were micromachined on a silicon or silicon-on-insulator wafer, which provided the required residual stress to shape cantilevers that bent out of the plane after releasing, such as silicon nitride [7,10,15], silicon dioxide [9,37] and an aluminum nitride/molybdenum multilayer [26].

In this section, we review the processes used to fabricate these three stressor layers (SiN, SiO${}_{2}$, AlN/Mo). Their manufacture can be subdivided into four main stages, namely: (1) depositing functional material layers to induce the required residual stress; (2) depositing piezoresistors and contact pads; (3) defining the cantilever shape; and (4) releasing the cantilever.

**Figure 1:**
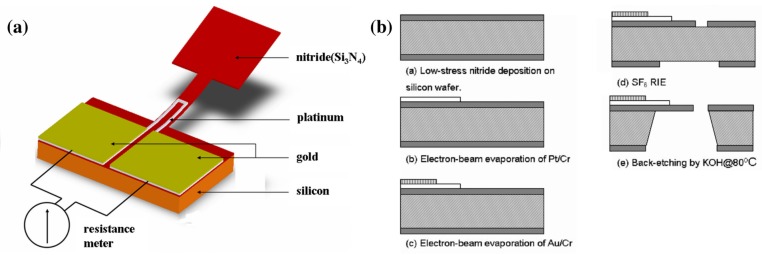
A MEMS-based air flow sensor with a free-standing micro-cantilever structure. (**a**) Schematic illustration and (**b**) overview of fabrication process employed for a gas flow sensor. Reproduced from Wang et al. [7].

Figure 1 gives an overview of the back-side bulk micromachining fabrication process for the MEMS-based air flow sensor described by Wang et al. [7]. Residual stress, released during the thermal fabrication process, was used to create a freestanding micro-cantilever beam, which used a platinum piezoresistor as the actual sensing element. Their fabrication process started with a low-pressure chemical vapor deposition of a 1 μm low-stress silicon nitride layer on either side of a silicon wafer (500 μm). In a subsequent electron-beam evaporation process, a thin chromium adhesion layer (20 nm) was deposited on the nitride layer. A second electron-beam evaporation process deposited the piezoresistor, a 100 nm platinum layer. Following the same deposition technique, a chromium adhesion (20 nm) and gold layer (400 nm) was deposited on top of the platinum layer to manufacture contact pads, the required electrical interface between the piezoresistor and an external resistance meter. Patterning the upper and lower nitride layers using Sulfur Hexafluoride (SF6) Reactive Ion Etching (RIE) plasma and, finally, releasing the cantilever structure by performing a potassium Hydroxide (KOH) back-etching process at 80 ${}^{\circ }C$, caused the cantilever structure to bend upward. By repeating the described MEMS procedure, but using different photomasks and layer thicknesses, Wang et al. [15] reassembled the air flow sensor with four cantilever beams positioned perpendicular to each other.

**Figure 2:**
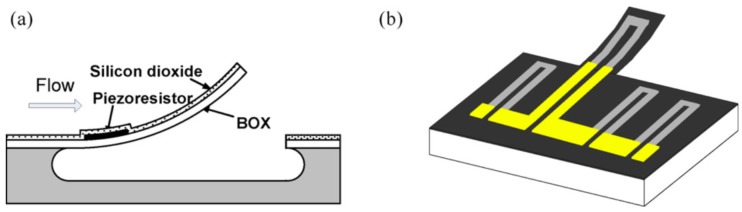
Self-bended silicon dioxide piezoresistive microcantilever flow sensor. (**a**) Cross-sectional and (**b**) perspective view. The highlight in (b) represents the metal interconnects. Reproduced from Zhang et al. [9] with permission of Elsevier B.V.

Zhang et al. [9] developed a top-side bulk micromachining releasing method using isotropic RIE to fabricate bent cantilevers on SOI wafers. The fabrication process was reported in detail by previous authors [9,37] and is introduced briefly here. Instead of using a nitride-based stressor layer as described by [7], their approach is based on two silicon dioxide layers deposited by using two different deposition techniques and temperatures, as shown in Figure 2. While the first 400 nm-thick Buried silicon dioxide (BOX) layer was fabricated using thermal oxidation at high temperatures above 1000 ${}^{\circ }C$, the second silicon dioxide layer (200 nm) was deposited by Plasma-Enhanced Chemical Vapor Deposition (PECVD) at a low temperature of 300 ${}^{\circ }C$. This temperature difference generated different residual stresses in the two silicon dioxide layers, which in turn curved the cantilever upwards after its release. The piezoresistor was sandwiched between the two silicon dioxide layers. Similar to the nitride-based process [7] cited earlier, the residual stress in the silicon dioxide-based process is very sensitive to deposition parameters and requires an elaborate operation of the MEMS manufacturing process.

**Figure 3:**
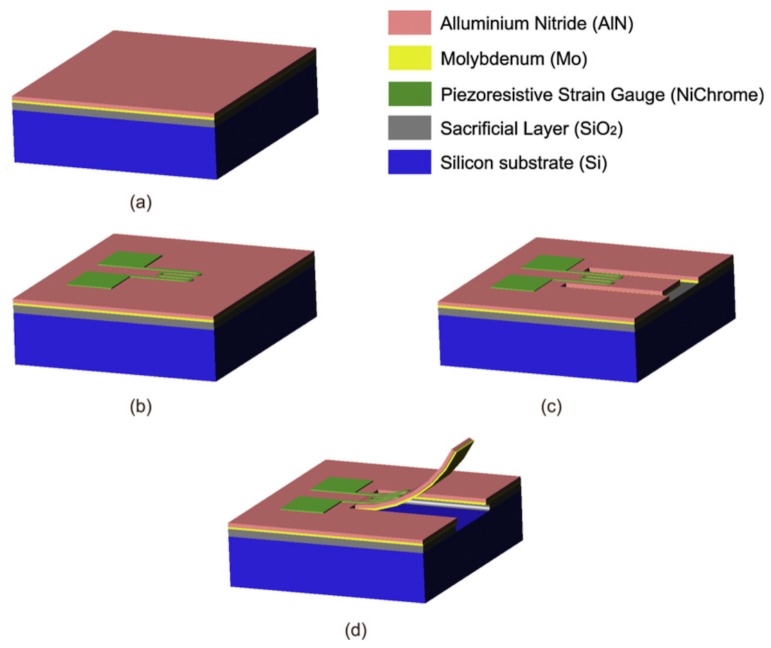
The MEMS fabrication process for an aluminum nitride/molybdenum based flow sensor. The process is subdivided into four main steps, that is (**a**) depositing functional material layers; (**b**) depositing piezoresistors and contact pads (thermal evaporation); (**c**) defining the cantilever U-shape (dry etching); and (**d**) releasing the cantilever (wet etching). Reproduced from Qualtieri et al. [26] with permission of Elsevier B.V.

Qualtieri et al. [26] described a surface micromachining fabrication process for AlN/Mo-based stress-driven micro-cantilevers, as illustrated in Figure 3. To induce residual stress, the top side of a silicon wafer (400 μm thickness) was coated with 200 nm silicon dioxide, 100 nm molybdenum and 600 nm aluminum nitride. A nichrome 80/20 (80% nickel, 20% chrome) piezoresistor (100 nm) was deposited using physical thermal evaporation. To improve adhesion, 10 nm chrome was deposited between the AlN layer and the piezoresistor. A third deposition process layered two 150 nm-thick gold contact pads. As before, 10 nm chrome between the nichrome and gold layer improved surface adhesion. To generate the U-shaped release pattern for the cantilever beam, Silicon tetrachloride (SiCl${}_{4}$)-based Inductively-Coupled Plasma (ICP) dry etching at the substrate top side patterned the first two layers and finished at the SiO${}_{2}$ layer. Designed as a sacrificial layer, the SiO${}_{2}$ layer beneath the cantilever beam was removed by isotropic wet etching in a hydrofluoric acid solution. Caused by the residual stress in the material, the released AlN/Mo bilayer bent out of the plane after the releasing process.

**Figure 4:**
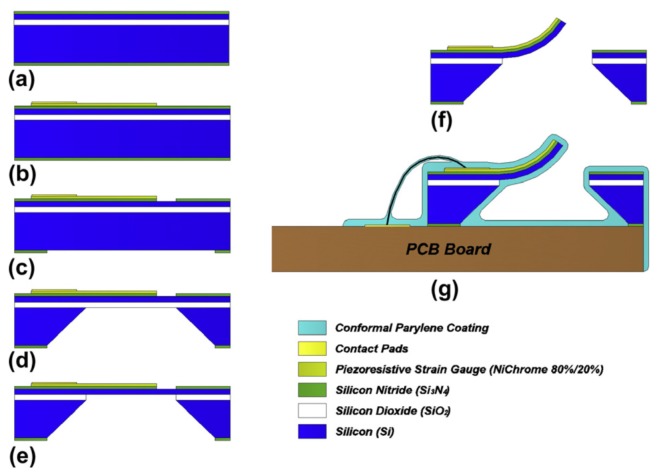
The MEMS fabrication process for a silicon nitride/silicon-based flow sensor. The process is subdivided into five main steps, that is: (**a**) depositing functional material layers; (**b**) depositing piezoresistors and contact pads (thermal evaporation); (**c**) defining the cantilever U-shape (dry etching); (**d**–**f**) releasing the cantilever (wet etching); and (**g**) waterproofing the sensor (chemical vapor deposition). Reproduced from Qualtieri et al. [38] with permission of Elsevier B.V.

Adapted from their AlN/Mo flow sensor, Qualtieri et al. [38] developed a second bulk micromachining process for a SiN/Si-based stress-driven microcantilever, as shown in Figure 4. An SOI substrate, made up of a 400 μm silicon wafer, a 2 μm-thick SiO${}_{2}$ insulation layer and a 2 μm silicon device layer, was coated with 300 nm SiN on the bottom and top sides. Four nichrome 80/20 piezoresistors (100 nm thick), distributed along the full length of the cantilever beam and arranged electrically into a Wheatstone-bridge circuit, were deposited using a physical thermal evaporation. To improve adhesion, 10 nm chrome was deposited between the SiN layer and the piezoresistors. A third deposition process layered four gold contact pads (150 nm) onto the nichrome layer with 10 nm chrome in between to improve adhesion. SiCl${}_{4}$-based ICP dry etching at the top side SiN layer was performed to generate the U-shaped release pattern for the cantilever. A second ICP etching process of the bottom side SiN layer was performed to open an aperture to the silicon substrate underneath the cantilever beam. Subsequently, anisotropic back side wet etching with a 28% KOH solution at 85 ${}^{\circ }C$ created a cavity beneath the cantilever beam. The SiO${}_{2}$ insulating layer acted as an etching barrier. Next, hydrofluoric acid back side wet etching removed the SiO${}_{2}$ layer. By performing a last KOH top side wet etching, the exposed (U-shaped) silicon layer around the cantilever was removed. Caused by the residual stress in the material, the released SiN/Si bilayer bent out of the plane after releasing. After bonding wires to the contact pads, a waterproof parylene coating by chemical vapor deposition was performed, which added a conformal 2 μm cover layer to all sides of the flow sensor (including the cantilever beam).

### 3.2. Microfabrication of Piezoelectric Tactile Membranes

In the past, the main microfabrication techniques for piezoelectric tactile sensors did not use any stress-driven building approach. The most commonly adopted designs for the realization of piezoelectric tactile sensors involved flat mechanical structures as pressure sensitive elements, typically sandwiched between electrodes [31,39]. Bump structures, usually made of silicone or soft materials like rubber, were then attached to the top of the piezoelectric elements to transfer the contact forces to the sensitive element.

An array of square flat elements of PZT, formed by sol-gel techniques with a thickness of ≈400 nm, fabricated on a thin elastomer substrate of silicone (thickness of 3.6 μm), were developed by Dagdeviren et al. [39] (as shown in Figure 13). Platinum electrodes were used to connect the PZT elements. Similarly, pressure sensors in the form of segmental arrays of parallel flat plate structures were fabricated by Khan et al. [31] sandwiching the piezoelectric P(VDF-TrFE) film between two printed metal layers of silver. Each module consisted of a 4×4 sensor array with a sensitive area of about $1\times 1\;mm^{2}$. The sensitivity of the final sensor was mostly determined by the piezoelectric properties of the material [33,40,41].

**Figure 5:**
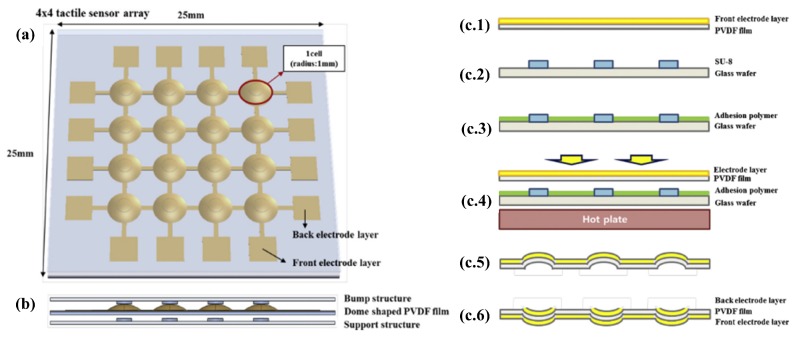
Tactile sensor array fabricated by inflation technique. Conceptual (**a**) front and (**b**) side views and (**c**) fabrication steps for the tactile sensor array. Reproduced from Kim et al. [30] with permission of Elsevier B.V.

Feng et al. [42] proposed the development of piezoelectric Dome-Shaped-Diaphragm Transducers (DSDTs) because 3D structures are able to achieve higher sensitivity than their planar counterparts by concentrating the applied pressure at the center of the piezoelectric curved cell. Afterwards Li et al. [29] realized this innovative 3D dome-shape P(VDF-TrFE) tactile sensor using a mold-transfer method from a cyclic-olifen-copolymer (COC) lens mold and standard MEMS techniques. To maintain the shape of the final sensor, the cavity of the domes was filled with implantable-grade silicone adhesive. Kim et al. [30] also demonstrated that a dome-shaped piezoelectric tactile sensor fabricated by an inflation technique can achieve higher sensitivity than the conventional flat structures. They conceptually fabricated the final sensor by trapping and inflating the air by means of a pre-polarized film of PVDF, a glass wafer and a face structure. A silver electrode layer (thickness = 10 μm) was deposited on a PVDF film by screen printing. Then, 250 μm-thick SU-8 structures were patterned on a glass wafer and used for local deformation of the PVDF film once the film is attached on the glass substrate. Through subsequent heat treatments, allowing the PVDF film to change shape, air was inflated and trapped underneath the locally deformed PVDF regions to achieve the desired dome geometry. The PVDF film was then released from the glass wafer. Finally, the PVDF film was turned upside down, and the back electrode layer was deposited by screen printing. Figure 5 displays the schematic illustration and the fabrication steps for the dome-shaped tactile sensors described by Kim et al. [30].

**Figure 6:**
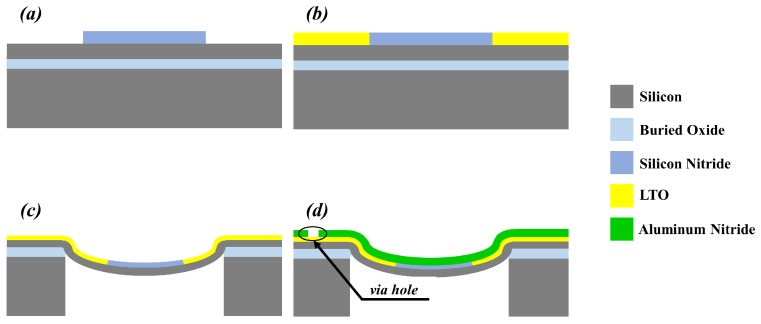
Cross-sectional view of the concave-shape pMUT (piezoelectric Micromachined Ultrasonic Transducer) generated by compressive residual stress of SiN and Low Temperature Oxide (LTO). (**a**) SiN deposition and patterning; (**b**) LTO deposition and Chemical Mechanical Polishing (CMP); (**c**) backside etching to form the diaphragm; (**d**) Mo/AlN/Mo deposition. Based on Akhbari et al. [43].

A stress-driven approach was implemented by Akhbari et al. [43]. They analytically and experimentally demonstrated improved electromechanical coupling properties of self-curved transducers, made of a 2 μm-thick aluminum nitride layer sandwiched between bottom and top metal electrodes, whose thickness was 150 nm, exploiting a self-generated curvature due to residual stress in the films. A multi-layer of 0.65 μm silicon nitride and Low Temperature Oxide (LTO) on top of 4 μm silicon has resulted in the desirable self-curved diaphragms by exploiting the residual compressive stress (≈180 MPa). Backside Deep Reactive Ion Etching (DRIE) was then used to release the self-curved diaphragm, which bends in a concave form. Finally, the active layers were deposited. The concave-shape diaphragm fabricated by Akhbari et al. [43] and the corresponding process flow are shown in Figure 6.

A different process was reported by Mastronardi et al. [27,28]. They designed and fabricated aluminum nitride-based domed circular membranes using the following standard microfabrication process. A Mo/AlN/Mo material stack was deposited on a general purpose Kapton HN${}^{\mathrm{TM}}$ (DuPont${}^{\mathrm{TM}}$) flexible substrate, previously laminated on a silicon wafer that acted as rigid support during the microfabrication steps. The AlN thin film, with a thickness of about 1 μm, was deposited at moderately high temperature (about 250–300 ${}^{\circ }C$) through reactive sputtering from a highly pure Al target. After a first masking photolithography, the top Mo and the AlN layers were etched into a circular shape by ICP etching. Next, the Mo bottom common electrode was patterned. In order to define the top electrodes, an additional metal deposition, followed by lift-off patterning was performed. Electrical connections were bonded in a subsequent step. Finally, to prevent external mechanical damages and to electrically isolate the piezoelectric transducers, a parylene C coating of 1 μm was deposited via chemical vapor deposition. The conformal nature of the parylene C coating allowed a full electrical and mechanical isolation of the final device.

## 4. Results and Applications

In this section, results and application examples of the previously described stress-driven MEMS structures for flow and tactile sensing are described. It will be shown that the stress-driven design or the ability to introduce change in the flexural bending stiffness of multilayered structures are the key points for setting up a specific mechanical behavior of the artificial sensor. A proper functional and morphological design combined with a detection principle (piezoresistive or piezoelectric) and the choice of the manufacturing strategy as described in the previous section is the way to achieve devices that are able to mimic and recreate the capabilities of biological sensors.

### 4.1. Piezoresistive Flow Sensing: Mimicking the Biological Lateral Line Organ

Over the course of evolution, fish have evolved a special sensory organ ideally adapted to their aquatic environment: the lateral line organ offers valuable information about the adjacent water movement by detecting changes in the pressure and water flow along the body. This so-called “distant touch” forewarns fish of predators, helps to avoid obstacles and may play a role in saving energy during underwater locomotion [44,45].

The individual sensory receptors in the lateral line organ, hair-like structures called neuromasts, detect the tiniest differences in pressure and perceive fractional changes of the flow velocity [46]. As a general functional principle, water flows around the neuromasts and bends their jellylike cupula protruding into the fluid. This flexible bending causes a mechanical deflection of the membrane cilia of hair cells, which are located inside the cupula, either leading to an excitation or an inhibition of a neuronal stimulus, depending on the direction of deflection.

From an engineering and biomimetics perspective, the implementation of an artificial lateral line system, inspired by how nature has solved the problem in fish, is a promising approach to explore hydrodynamics and to test the hypothesis that lateral line sensing can improve the propulsion efficiency, sensing capability and feedback control systems in underwater vehicles.

#### 4.1.1. Bio-Inspired Artificial Hair Cells

Artificial lateral line flow sensing applications require hair-like structures with a length enough to reach into the laminar flow layer above the vehicle’s skin. Production processes employed in microsystems technology allow for sufficient miniaturization of the components required to develop bio-inspired artificial hair cells with the required dimensions. Various piezoresistive-based sensor design methodologies and MEMS fabrication processes for producing hair cell-like flow sensors have been developed in various laboratories world-wide, as thoroughly described in several comprehensive literature reviews [47,48,49,50,51]. Table 1 gives a comparative overview of piezoresistive-based sensor design methodologies by listing achieved hair geometries, aspect ratios and flow velocity-related performance indicators. Early successes were achieved with bare vertical beams and pillar structures. Subsequent improvement in the available manufacturing processes has made it possible to implement various microstructures, such as dome-like cupulae, pillars capped with hydrogels, bent cantilevers and flags and, most recently, interconnected carbon nanotube bundles [52].

**Figure 7:**
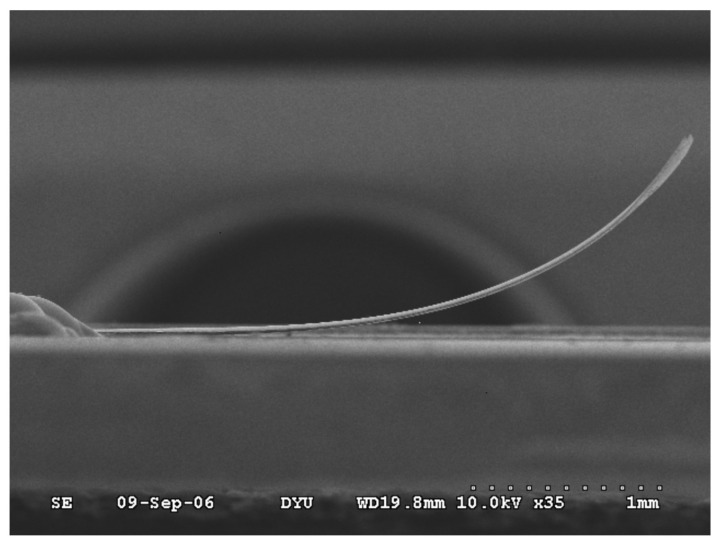
Side view scanning electron microscope image. MEMS-based air flow sensor with a free-standing microcantilever structure. Reproduced from Wang et al. [7].

Wang et al. [7] performed a systematic investigation to characterize their MEMS-based air flow sensor, as shown in Figure 7. Tests with three different cantilever beam lengths (400 μm, 1200 μm and 2000 μm) were performed in a wind tunnel at airflow velocities ranging between 0 and ≈45 $m\;s^{-1}$ (maximum detectable flow rate). The resistance signal generated by the flow sensors increased approximately linearly with increasing airflow velocity, and flow rate sensitivity increased with increasing cantilever beam widths. Average sensitivities were found to be 0.0134, 0.0227 and 0.0284 $\Omega \;m^{-1}s$, respectively.

**Figure 8:**
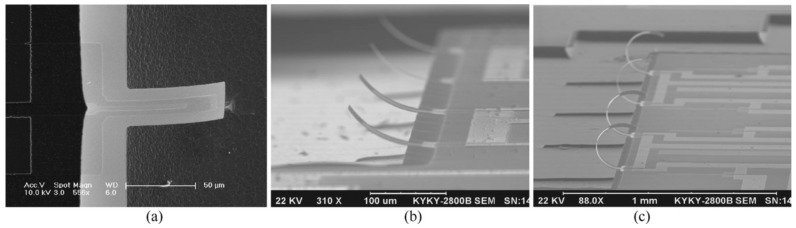
Scanning electron microscope images of curved-up microcantilever flow sensors. (**a**) Single 100 μm microcantilever, (**b**) 100 μm microcantilever array and (**c**) 400 μm microcantilever array. Adapted from Zhang et al. [9] with permission of Elsevier B.V.

Zhang et al. [9] used deionized water to calibrate their self-bended piezoresistive microcantilever flow sensors. Micro-cantilevers with beam lengths of 100 μm, 200 μm and 400 μm were fabricated, as shown in Figure 8. Varying flow rates between of 0 and 0.2 $m\;s^{-1}$ were applied to test the performance under controlled conditions. The results revealed that the microcantilever was able to measure small flow rate between 0 and 0.23 $m\;s^{-1}$, with a sensitivity ranging between 1.5 and 3.5 $\Omega \;{\mathrm{cm}}^{-1}s$.

**Figure 9:**
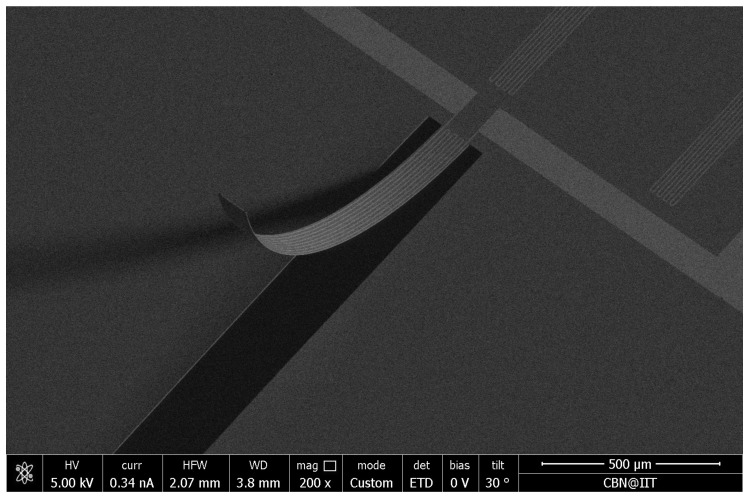
Scanning electron microscope image of the silicon nitride-based cantilever at 200× magnification. The released SiN/Si bilayer is 2.3 μm thick and reaches approximately 1.2 mm tip height above the base layer.

**Figure 10:**
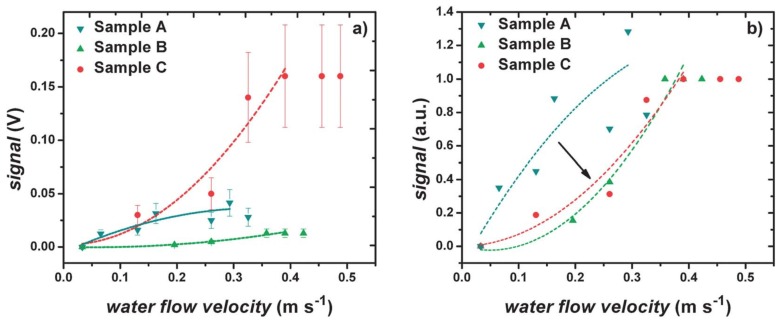
Electrical behavior of three flow sensors with varying material thicknesses under continuous water flow conditions. The 0.5 μm parylene coating (Sample A), 2 μm parylene coating (Sample B) and 0.3 μm Si${}_{x}$N${}_{y}$ layer (Sample C). The dashed lines are intended as a visual guideline. (**a**) The different coating material characteristics result in varying calibration curve shapes, tuning from a sub-linear (strain-hardening) to a super-linear (strain-softening) trend. A common signal saturation region is shown. (**b**) Normalized output signal: the arrow highlights the different behaviors of the calibration curve, going from lower to higher flexural stiffness. Reproduced from Rizzi et al. [14] with permission of The Royal Society of Chemistry.

The stress-driven artificial hair cell described by Qualtieri et al. [38] was tested and calibrated in continuous water flow up to 0.5 $m\;s^{-1}$. A scanning electron microscope image of the artificial hair cell is presented in Figure 9. The SiN/Si-based cantilever reaches approximately 1.2 mm tip height above the base layer. A thin hydrophobic parylene layer covers and securely waterproofs the entire surface of the sensor [14,38]. Figure 10 shows the electrical behavior of the flow sensor with varying material thicknesses in a continuous water flow. The relative sensor signal is plotted as a function of water flow velocity. The varying curve shapes demonstrate that the sensitivity of the flow sensor to a specific dynamic range can be tuned by choosing the parylene thickness accordingly. Different material thicknesses resulted in varying beam flexural stiffnesses, which in turn, generated varying signal amplitudes. Sample A (parylene coating, 0.5 μm) showed a sub-linear (strain-hardening) behavior with a linear sensitivity of ≈0.2 $V\;m^{-1}\;s$ at flow velocities lower than 0.20 $m\;s^{-1}$. In comparison, Samples B (parylene coating, 2 μm) and C (Si${}_{x}$N${}_{y}$ layer, 0.3 μm) showed a super-linear (strain-softening) behavior with a linear sensitivity of ≈0.07 and 0.9 $V\;m^{-1}\;s$, respectively, at higher flow velocities between 0.25 and 0.35 $m\;s^{-1}$.

#### 4.1.2. Artificial Lateral Line Flow Rate and Velocity Sensing

**Figure 11:**
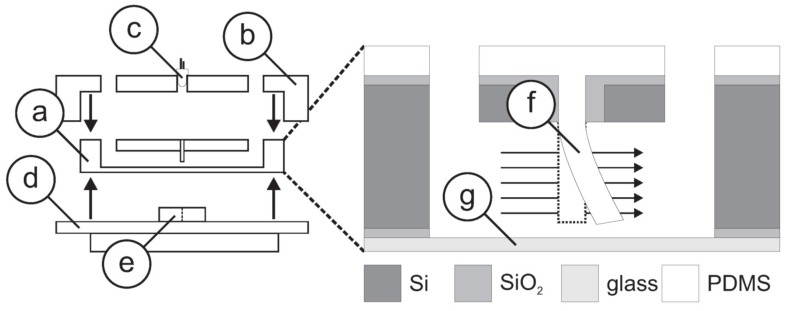
Assembly of a micro-machined optical-based flow sensor. (**a**) Si-chip; (**b**) housing; (**c**) LED; (**d**) electronics PCB; and (**e**) optical detector. Magnification: Si-chip featuring a (**f**) PDMS lamella and (**g**) glass plate. Dimensions not to scale. Reproduced from Herzog et al. [66].

Klein and Bleckmann [65] and Herzog et al. [66,67] described a bio-inspired flow rate sensor that uses an optical measuring method to be applied in tap water systems and medical and pharmaceutical applications. As shown in Figure 11, a single artificial flow sensor was embedded in a canal and consisted of a lamella. An LED was attached above the canal and coupled light into the lamella, which in turn was converted into electricity by a photodiode positioned at the bottom side. When water streamed in the canal, the lamella either deflected by a constant angle causing a steady signal (in case of steady laminar flow) or the vibrations of the lamella caused an unsteady, vibrating and oscillating signal (in case of turbulent flow with fluctuations and unsteadiness).

Wang et al. [15] positioned four freestanding micro-cantilever beams (as described in Section 3.1) perpendicular to each other to detect flow rate and direction. When air propagated through the sensor array, the resulting beam deformation caused resistance variation among the individual cantilever beams (4000 μm long and 400 μm wide), which was used to determine air flow direction. When the air propagated through the sensor array in parallel to two opposing beams, the largest resistance variation was found for the downwind cantilever, while the least resistance variation was caused by the upwind cantilever. The resistance variations of the two cantilever beams positioned perpendicular to the air flow direction were almost equal to zero. Furthermore, it was shown that flow rate can be determined by calculating the total resistance variations for the four cantilevers. For two given flow directions (i.e., 135° and 180° as shown in [15]), the sum of the absolute values of the resistance variation was equal and just depended on the flow rate.

**Figure 12:**
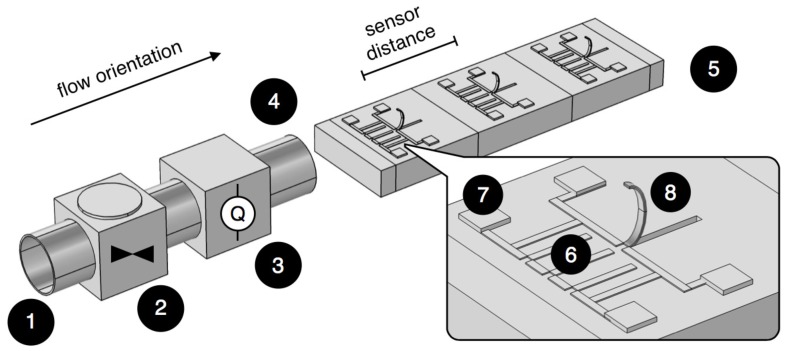
Simplified systematic overview of the experimental setup. Flow pulses pass the following components: (1) non-flexible tubing; (2) mechanical valve; (3) commercial flow rate sensor; (4) tube outlet; and (5) artificial lateral line system with artificial hair cell sensors positioned in a line. Magnification: (6) piezoresistors; (7) contact pads; and (8) cantilever beam. Flow orientation and sensor distance are indicated. Dimensions not to scale. Reproduced from Abels et al. [68] with permission of IOP Publishing.

In the flow sensing application described by Abels et al. [68], a linear array of closely separated stress-driven artificial hair cells was designed that features multi-parameter flow measurements to be used as input for an underwater vehicle’s control procedure. The flow sensing array consisted of multiple flow sensors in a line along the cantilever beam direction. Figure 12 gives a simplified systematic overview of the experimental setup. A real-time capable cross-correlation procedure was developed, which extracts freestream flow direction and velocity information from flow fluctuations. When flow fluctuations (or pulses) propagated through the sensor array, similar, but time-shifted flow signals were detected by the individual sensors. By cross-correlating multiple sensor signals, relevant information about local flow velocities in the sensor array, as well as propagation velocity, linear forward/backward direction along the cantilever beam orientation and periodicity of pulses or pulse trains was extracted. In general, flow velocity information was in strong agreement with a commercial system. The computed flow velocities deviated from the commercial system by 0.09 $m\;s^{-1}$ for 0.5 $m\;s^{-1}$ flow velocity and by 0.15 $m\;s^{-1}$ for 1.0 $m\;s^{-1}$ flow velocity. In case very high accuracy is required by the technical application, precise information about velocity components of the flow can be computed for signal-to-noise ratios down to about 2.5 or five, in case of filtered or unfiltered signals, respectively.

### 4.2. Piezoelectric Tactile Sensing: Mimicking the Human Tactile Sense

The human tactile sense is an extremely accurate and sensitive active organ. It is responsible for detecting mechanical stimuli in a pressure range from 10–100 kPa, in addition to thermal and other stimuli, that typically occur during the dexterous manipulation of objects, providing information about contact forces, distribution and torques and allowing for the identification of object properties, such as geometry, stiffness and texture [69,70]. Tactile receptors are usually classified as mechanoreceptors, being able to convert the mechanical deformations caused by forces, vibrations or slip of the skin and objects into electrical nerve impulses. When the skin is deformed, the corresponding deformation is transmitted from the surface to the mechanoreceptor’s plasma membrane causing the generation of a graded potential and, in turn, corresponding spikes of action potentials [71,72]. Human skin and especially the fingertips contain different types of tactile sensors, which can be categorized according to the nature of the stimulus they detect: static or dynamic mechanoreceptors. While static receptors are sensitive to temporally constant pressure, dynamic sensors are responsive to time-variant stimuli [73,74].

From an engineering point of view, humanoid robots need tactile interfaces to perform complex tasks in unstructured environments and for safely interacting with humans in daily and routinely executed activities, such as assistance, support and coexistence. Bio-mimicking abilities of the human tactile system and performing advanced in-hand manipulation tasks are made possible by detecting static forces, as well as dynamic forces, such as normal and tangential contact for gathering spatial and geometrical information from surface exploration. Arrays of pressure-sensitive sensors could be integrated into an electronic skin to complement the desirable properties of flexibility, conformability and stretchability in improving anthropomorphism [75,76].

#### 4.2.1. Tactile Sensors

In the last decade, the development of flexible materials for tactile sensing applications has attracted an increasing interest in robotics, industrial and manipulation applications, prosthetic and orthotic devices and tools for the evaluation of tissue stiffness and for preventing damage [29,30,31,33,39,40]. Achieving high spatial resolution and reliability by means of a large-area artificial skin-like sensory system is a priority for the enhancement of robots’ capabilities and their safe interaction with humans and objects with sufficient sensitivity in a wide pressure range (10–100 kPa). In this regard, aluminum nitride (AlN), a wide band-gap piezoelectric material, stands out as a useful candidate for artificial skin applications. Advantages such as low actuation voltages (1–10 V) and easy integration on CMOS technology and flexible substrates are key features of this technology.

Piezoelectric micromachined transducers are of great importance as measurement tools for pulse-echo ultrasonics applications in robotics, medical diagnostic and proximity detection [77,78,79,80]. Here, the ultrasonic transducer, behaving as a transmitter/receiver, is typically embedded in a soft material layer that can be deformed by an applied pressure. The pulse generated by the transmitter and propagating through the soft covering is reflected back by the target and collected by the same piezoelectric transducer (the receiver), such that it is possible to know the transit time of the signal, which is proportional to the thickness of the deformed material and, hence, to the applied load. Contact parameters can be detected also by measuring the change in resonance frequency of the sensor, allowing the detection of the hardness and/or compliance of objects. Flexure modes of piezoelectric membranes can be exploited to cover ultrasonic frequency ranges that extend from 100 kHz–50 MHz.

**Figure 13:**
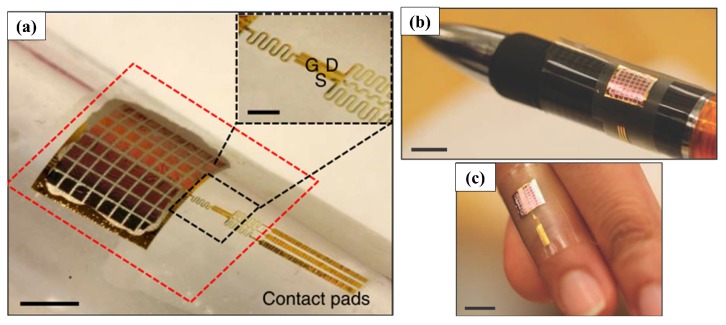
Photographs of a thin conformable piezoelectric pressure sensor. (**a**) Device wrapped on a cylindrical glass support (scale bar, 5 mm); (**b**) sensor wrapped on a pen; and (**c**) a finger (scale bar, 10 mm). Reproduced from Dagdeviren et al. [39] with permission of Macmillan Publishers Limited.

Using flat tactile pressure sensors that exploit the dependence of the piezoelectric voltage on the contact force, Dagdeviren et al. [39] (as shown in Figure 13) used an array of piezoelectric square membranes to make a precision skin-mounted sensor to measure pressures in a very low load range from 2–10 mN with a dynamic sensitivity of about 11.6 $mV\;N^{-1}$ and a minimum detected force of ≈2 mN (see Table 2). Khan et al. [31] exploited the changes in the polarization level of the P(VDF-TrFE) to measure the contact forces and detect dynamic tactile parameters. The array was able to detect contact forces in a range of [400–4000] mN with a dynamic sensitivity of ≈500 $mV\;N^{-1}$ and a minimum detected force of 200 mN.

**Figure 14:**
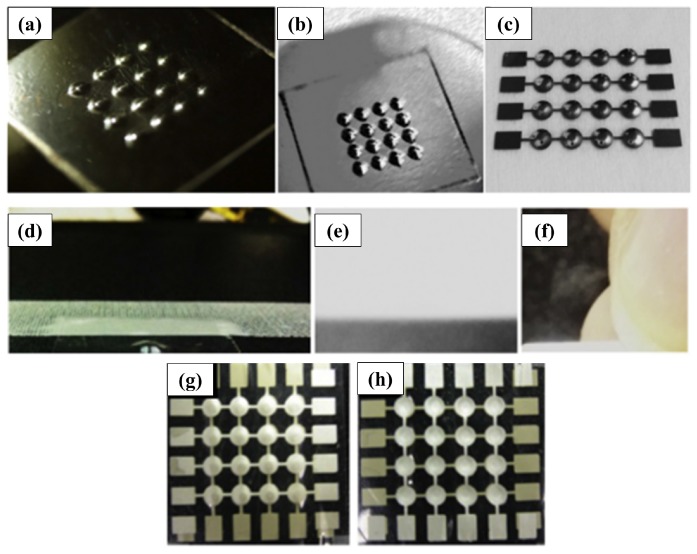
Optical photographs of the fabricated dome-shaped PVDF film and tactile sensors. (**a**–**f**) The fabricated dome-shaped PVDF film (height = 500 μm). (**g**,**h**) The proposed dome-shaped tactile sensor. Reproduced from Kim et al. [30] with permission of Elsevier B.V.

An improvement in the sensitivity of piezoelectric tactile sensors can be achieved by adding complexity to the geometry of the sensor. Kim et al. [30] fabricated a dome-shaped piezoelectric tactile sensor (Figure 14) with films of 56 μm-thick PVDF whose achieved sensitivity can reach values up to 8830 $mV\;N^{-1}$ (according to the geometry and curvature of domes), showing an increase of about 46% of the sensitivity compared to the conventional flat counterpart.

**Figure 15:**
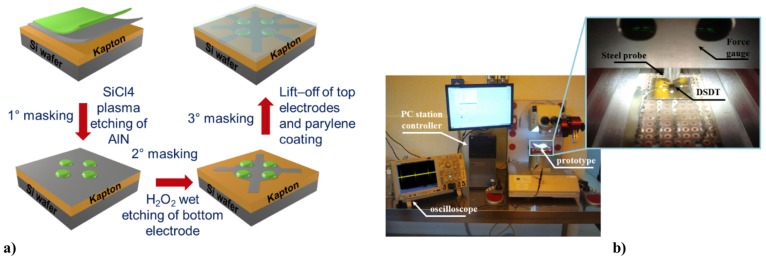
An array of 2×2 aluminum nitride-based tactile transducers. (**a**) Dome-shaped diaphragm transducers have been designed and realized by a standard micromachining process; (**b**) computer and probe station used for measurements and calibration. Reproduced from Mastronardi et al. [27] with permission of AIP Publishing.

As illustrated in Figure 15, an array of 2×2 aluminum nitride-based tactile transducers, hereafter named DSDT (Dome-Shaped Diaphragm Transducer), was designed and manufactured using a standard micromachining process on 25 μm-thick polyimide flexible substrate (see Section 3.2) [27,81,82]. The unit cell consists of flexible piezoelectric circular membranes made of c-axis highly-oriented AlN embedded between Mo metal electrodes in order to collect the charges generated by virtue of the direct piezoelectric effect. The stress release of the active layers (AlN and Mo marginally) generates uplifted and circular domes with structural stiffness well below the stiffness of the component materials (up to 3.5 $N\;mm^{-1}$). The overall effect is an improvement of the electromechanical response of the piezo-cells: the lower the stiffness of the dome as a whole, the lower the minimum threshold for contact force detection and the larger the overall dynamic range of detection. The uplifted area is thus determinant for the low stiffness and dynamic range of the transducer that easily deforms under loading. The natural organization in the dome structure is due to the stress difference of AlN over the polyimide, which leads to a lifting up of the circular structures from the substrate surface. The sensing mechanism of the transducer mainly relies on the dome shape; when a normal force is applied on the top of the dome, a mechanical stress occurs in the AlN thin layer that becomes electrically polarized due to synergistic interaction between piezoelectric and flexoelectric effects, making possible the measurement of periodic impulsive mechanical stimuli. The domes are first pushed downwards, with slight compression in the softer polyimide substrate tape. Then, the released structure is flattened on the silicon substrate. In this region, the stress is transferred to the piezoelectric film, generating the voltage signal, that goes to saturation caused by a constant flattening of the active piezoelectric layer. The dielectric properties of the MIM (Mo/AlN/Mo) stack, which behaves as a capacitor, also make possible the measurement of long time frame static mechanical stimuli by a steady deformation of the convex structure [27].

#### 4.2.2. Tactile Sensing System

**Figure 16:**
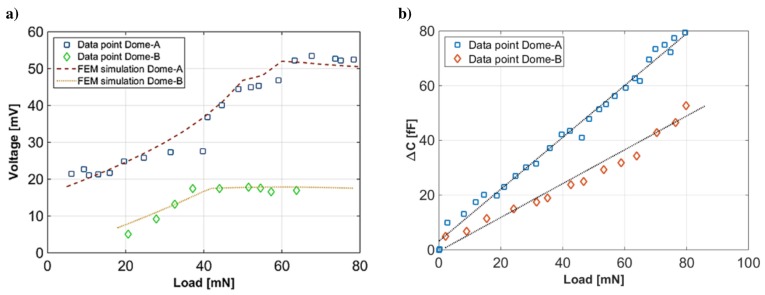
Electrical characterization of dome-shaped devices with different releasing heights. (**a**) The output voltage at the peak is reported vs. the applied force (Dome A, height h≈ 43 μm, uplifted radius $r_{up}\approx$ 1.5 mm and stiffness $k_{s}\approx$ 3.5 $N\;mm^{-1}$; Dome B, height h≈ 33 μm, uplifted radius $r_{up}\approx$ 1.2 mm and stiffness $k_{s}\approx$ 9.7 $N\;mm^{-1}$); (**b**) the detection of long static stimuli has been experimentally and analytically observed as capacitance decrease at increasing forces up to 80 mN of contact force. Reproduced from Mastronardi et al. [27] with permission of AIP Publishing.

Dome-shaped devices with different releasing height (Dome A, height h≈ 43 μm, uplifted radius $r_{up}\approx$ 1.5 mm and stiffness $k_{s}\approx$ 3.5 $N\;mm^{-1}$; Dome B, height h≈ 33 μm, uplifted radius $r_{up}\approx$ 1.2 mm and stiffness $k_{s}\approx$ 9.7 $N\;mm^{-1}$) were electrically characterized, as reported in Figure 16, where the output voltage at the peak is reported vs. the applied force (Figure 16a). As can be seen from the plots in Figure 16a,b, the electromechanical response of the domes is related to the radius of the uplifted area: the larger the radius of the outer dome area (the region not covered by piezoelectric material), the higher the sensitivity of the sensor to lower forces because of the combination of piezoelectric and flexoelectric effects. On the other hand, domes with smaller uplifted area have shown lower measured offset, because of a less significant flexoelectric contribution. Measurements show an increase of generated voltage in a dynamic range of [0–60 mN]. For higher applied forces, the devices start to saturate. Nevertheless, the minimum force detected is 1.2 mN. The detection of long static stimuli (Figure 16b) was experimentally and analytically observed as a capacitance decrease at increasing contact forces of up to 80 mN. The capacitance variation was observed for the whole interval of application of the stress (1 s), and it did not decay with time; therefore, the measurement of static forces is possible in the same system, making the AlN thin film in the dome structure a multifunctional material.

**Figure 17:**
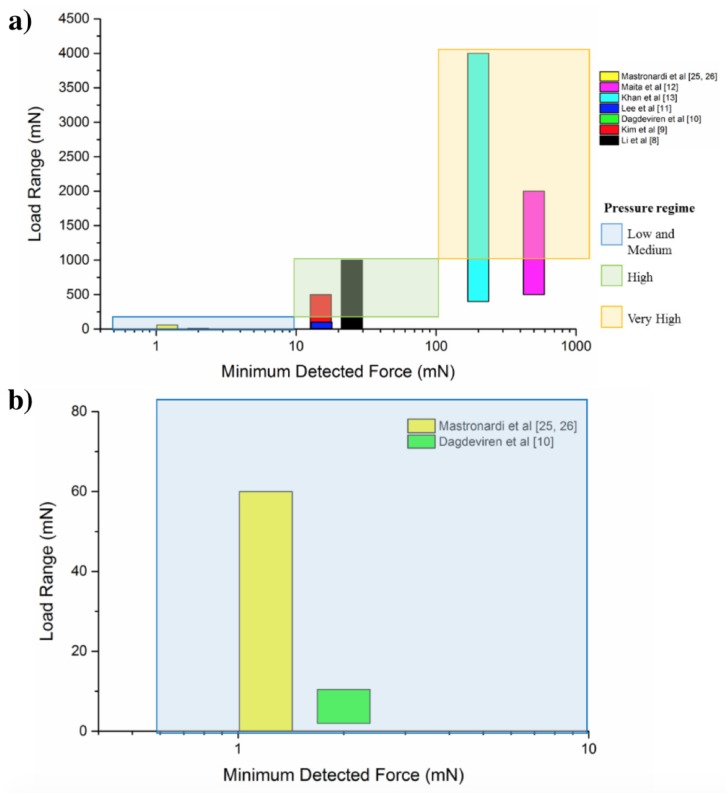
Pressure detection in the low and medium, high and very high pressure regime. (**a**) Mapping of the listed devices (Table 2) to their detectable load ranges; (**b**) magnification of the low and medium pressure regime.

An exhaustive comparison of the technologies based on aluminum nitride and other different approaches, still based on piezoelectric transduction methods, for the development of tactile sensors is reported in Table 2, and the comparison is displayed graphically in Figure 17.

As shown in Table 2, the integration of domed structures made of aluminum nitride and flexible substrate is a strategy to have both dynamic and static detection of contact forces and pressures. By considering the surface area of a single DSDT, about 0.384 $\mu m^{2}$, it can be assessed that the AlN and polyimide-based sensors successfully detect pressure from the low-pressure regime (1 kPa–10 kPa) up to the medium-pressure regime (10 kPa–100 kPa), as illustrated in Figure 17.

Aluminum nitride was successfully used to develop and fabricate piezoelectric micromachined ultrasonic transducers. Akhbari et al. [43,77] demonstrated the effects of the residual stress in AlN on the dynamic responses of stress engineered curved pMUT devices. Especially, as the residual stress in the AlN increases, the low-frequency displacement per unit input voltage drops, and the resonant frequency increases. Table 3 gives a comparative overview of pMUTs based on AlN active thin film, which were patterned with different shapes and geometries, moving from standard flat membranes, to flexurally suspended circular elements, up to concave or dome-shaped curved cells. The performance of each device, that is resonance frequency, displacement range and corresponding driving voltage, is listed.

**Figure 18:**
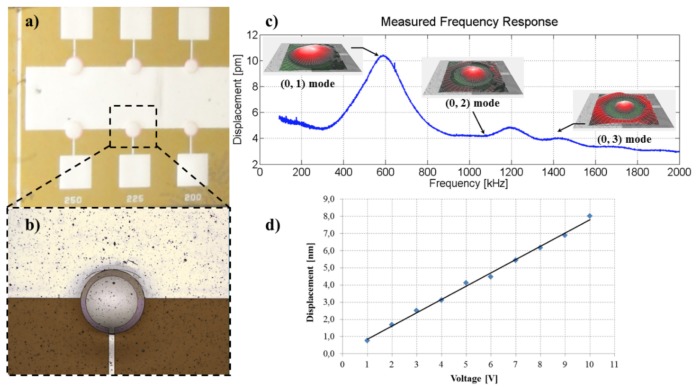
Mechanical behavior of the circular membranes of the final piezoelectric ultrasonic transducer. (**a**,**b**) Microscope pictures of the final piezoelectric ultrasonic transducer. (**c**) The mechanical behavior of the circular membranes has been investigated by driving the piezoelectric film by means of a sinusoidal voltage whose frequency sweeps from 0.5 Hz–2 MHz. Different mode shapes at resonances have been clearly identified. (**d**) The displacement amplitude increases linearly with actuation voltage. Reproduced from Mastronardi et al. [28] with permission of Elsevier B.V.

As reported by Mastronardi et al. [28], a pMUT consisting of flexible piezoelectric membranes of AlN (with a thickness of 800 nm) is fabricated on 25 μm-thick Kapton flexible substrate by exploiting the same technology of tactile sensors and the inverse piezoelectric effect. The final transducers (shown in Figure 18a,b) have a radius of 250, 275 and 300 μm. They were assumed to behave like mechanically-clamped homogeneous plate resonators by virtue of the three-dimensional structures achieved after the release of the residual stress (estimated as −48.1±0.5 MPa [28]).

The mechanical behavior of the circular membranes was investigated by driving the piezoelectric film by means of a sinusoidal voltage whose frequency sweeps from 0.5 Hz–2 MHz, as presented in Figure 18c. In this frequency range, the different mode shapes at resonances have been clearly identified. A typical Gaussian-like shape can be seen by the 3D reconstruction of the out-of-plane deflection of the (0, 1) fundamental resonant mode (inset in Figure 18c), where the maximum displacement is achieved at the center of the membranes. A maximum displacement of about 8.0 nm has been measured by driving the membranes with a maximum voltage of 10 $V_{\mathrm{pp}}$ (see Figure 18d), even if the membranes are still attached to a rigid support. Moreover, the amplitude of the displacement increases linearly with the actuation voltage, and no deviation from the linearity has been observed up to the maximum limit voltage of 10 $V_{\mathrm{pp}}$.

## 5. Conclusions

In this review, we described a stress-driven approach to design and fabricate flexible nitride-based MEMS devices for sensing applications. A controllable stress gradient along the cross-section of thin aluminum nitride and silicon nitride layers can be induced by appropriately designing and micromachining multilayered thin films. By depositing piezoresistive nichrome strain gauges onto nitride-based multilayers or by using direct piezoelectric properties of aluminum nitride-based microstructures, highly sensitive mechanical strain/stress detection can be realized, turning dome-shaped membranes and upwards-bent cantilevers into tactile and flow sensors for various fields of applications. Future development on stress-driven bio-inspired technology will be directed towards large area systems for robotics and ultrasonics applications. Next generation artificial hair cell-like flow sensors might not only approximate the morphological properties of biological neuromasts more precisely, but also bio-mimic physiological functions, such as bidirectional sensitivity and interconnections between individual hair cells. Dome-shaped devices could be designed as an array of sensors and ultrasonics generators for both dynamic and static tactile sensing based on contact and ultrasonic range finding. Future developments will be focused on the experimental evaluation of the contribution of flexoelectricity in a tactile stress-driven device as a function of the AlN material thickness, in order to better control flexoelectric properties.

## Figures and Tables

**Table 1:** Previously described piezoresistive based hair cell-like flow sensors ordered by year of publication. Comparative overview of hair geometries, aspect ratios and flow velocity related performance indicators (sensitivity, measurement range).

Authors	Hair Geometry ^a^	Aspect Ratio	Performance ^b,c^
Ozaki et al. (2000) [53]	Vertical beam (3000×250×8 μm)	12:1	n/a
Ozaki et al. (2000) [53]	Vertical pillar (800×230×10 μm)	3.5:1	n/a
Fan et al. (2002) [54], Chen et al. (2003) [55]	Vertical beam (820×100×10 μm)	8.2:1	n/a
Engel et al. (2005) [56]	Vertical pillar (3000×500 μm)	6:1	S = 245 ppm/μm
Tucker et al. (2006) [57], Chen et al. (2007) [58]	Vertical pillar (600×80 μm)	7.5:1	S${}_{water,AC}$ = 200 $\mu m\;s^{-1}$ ${}_{water,DC}$ = 100 $\mu m\;s^{-1}$
Peleshanko et al. (2007) [59]	Dome-like cupula (750×1500 μm)	1:2	S${}_{water}$ = 75 $\mu m\;s^{-1}$
Wang et al. (2007) [7]	Bent cantilever (4000×400×1 μm)	10:1	S${}_{air}$ = 0.0284 Ω/( $m\;s^{-1}$) R${}_{air}$ = 0–45 $m\;s^{-1}$
Wang et al. (2008) [15]	Bent cantilever (4450×200×20 μm)	22.3:1	n/a
Aiyar et al. (2009) [60]	Bent flag (1500×400×7.6 μm)	3.8:1	S${}_{air}$ = 66 Ω/( $m\;s^{-1}$) R${}_{air}$ = 0–16.9 $m\;s^{-1}$
Du et al. (2009) [61,62]	Cantilever (500×500×10 μm)	1:1	S${}_{air}$ = 60 μV/( $m\;s^{-1}$)
McConney et al. (2009) [63]	Capped vertical pillar (825×165 μm)	5:1	S${}_{water,bare}$ = 100 $\mu m\;s^{-1}$ S${}_{water,capped}$ = 2.5 $\mu m\;s^{-1}$
Song et al. (2009) [64]	Bent flag (3500×600×8.2 μm)	5.8:1	S${}_{air}$ = 14.5 mV/( $m\;s^{-1}$) R${}_{air}$ = 0–12 $m\;s^{-1}$
Zhou et al. (2009) [37], Zhang et al. (2010) [9]	Bent cantilever (100×20×1 μm)	5:1	S${}_{water}$ = 1.5–3.5 Ω/( $cm\;s^{-1}$) R${}_{water}$ = 0–0.23 $m\;s^{-1}$
Qualtieri et al. (2011) [26]	Bent cantilever (600×100×0.7 μm)	6:1	n/a
Qualtieri et al. (2012) [38]	Bent cantilever (1500×100×4 μm)	15:1	S${}_{water}$ = 0.7 mV/( $cm\;s^{-1}$) R${}_{water}$ = 0.05–0.35 $m\;s^{-1}$
Yilmazoglu et al. (2016) [52]	Vertical beam (500×350×100 μm)	1.4:1	S = 2100 ppm/μm

^a^ Hair geometry defined as product of length × width (or diameter) (×thickness); ^b^ R = dynamic range, S = sensitivity, n/a = not available; ^c^ ppm/μm = resistance change in parts per million per μm of tip deflection.

**Table 2:** Comparison between different technological approaches for force detection in tactile applications.

Authors	Material	Shape	Spatial Res. ^a^ (mm)	Min. Force (mN)	Dynamic Sensitivity (mV N^−1^)	Static Sensitivity (fF N^−1^)	Load Range (mN)	Voltage Output (mV)
Li et al. (2008) [29]	PVDF-TrFE	Dome	0.5	25	up to 10.6	/	0–1000	0–11
Kim et al. (2014) [30]	PVDF	Dome	0.9	15	up to 8830	/	0–500	0–5000
Dagdeviren et al. (2014) [39]	PZT	Flat	0.25	2	11.6	/	2–10.5	0.001–0.1
Lee et al. (2014) [40]	PZT	Flat	3	15.2	105	/	10–100	1–12
Khan et al. (2015) [31]	PVDF-TrFe + MWCN	Flat	/	200	500	/	400–4000	4–16
Maita et al. (2014) [33]	AlN	Flat	/	500	13	/	500–2000	3–10
Mastronardi et al. (2014, 2015) [27,82]	AlN	Dome	0.75	1.2	up to 480	up to 950	0–60	0–37

^a^ Res. = Resolution

**Table 3:** Comparison between pMUT made of AlN active thin-film.

Authors	Material	Shape	Radius (μm)	Resonance Frequency (kHz)	Displacement (nm)	Driving Voltage (V)
Shelton et al. (2009) [83]	Si/SiO${}_{2}$/AlN	Flat circular	175–225	220	1000–1300	0.5–7
Przybyla et al. (2010) [84]	Si/SiO${}_{2}$/AlN	Flat circular	200	214	100–750	0.5–15
Akhbari et al. (2014) [77]	Si/AlN	Concave curved circular	60–95	500–2190	0–5	0–10
Guedes et al. (2011) [85]	Si/SiO${}_{2}$/AlN	Flat flexurally suspended circular	200	121.3	0–1100	0–30
Mastronardi et al. (2014) [28]	PI/AlN	Dome curved circular	250–300	390–680	0.5–8	0–10

## Abbreviations

The following abbreviations are used in this manuscript:

AlN	Aluminum Nitride
CCC	Cross-Correlation Coefficient
CCF	Cross-Correlation Function
CMOS	Complementary Metal-Oxide-Semiconductor
CMP	Chemical Mechanical Polishing
COC	Cyclic Olefin Copolymer
CVP	Chemical Vapor Deposition
Cr	Chrome
DRIE	Deep Reactive Ion Etching
DSDT	Dome-Shaped Diaphragm Transducer
ICP	Inductively Coupled Plasma
KOH	Potassium Hydroxide
LPCVD	Low Pressure Chemical Vapor Deposition
LTO	Low Temperature Oxide
MEMS	Micro-Electro-Mechanical Systems
Mo	Molybdenum
PECVD	Plasma-Enhanced Chemical Vapor Deposition
pMUT	Piezoelectric Micromachined Ultrasonic Transducers
PVDF	Polyvinylidene Difluoride
PZT	Lead Zirconate Titanate
RIE	Reactive Ion Etching
SF6	Sulfur Hexafluoride
Si	Silicon
SiCl${}_{4}$	Silicon Tetrachloride
SiN	Silicon Nitride
SiO${}_{2}$	Silicon Dioxide
SOI	Silicon-On-Insulator
TrFE	Trifluoroethylene
UT	Ultrasonic Transducer
VDF	Vinylidene Fluoride
ZnO	Zinc Oxide
